# Adrenomedullin and tumour microenvironment

**DOI:** 10.1186/s12967-014-0339-2

**Published:** 2014-12-05

**Authors:** Ignacio M Larráyoz, Sonia Martínez-Herrero, Josune García-Sanmartín, Laura Ochoa-Callejero, Alfredo Martínez

**Affiliations:** Oncology Area, Center for Biomedical Research of La Rioja CIBIR, C/Piqueras 98, Logroño, 26006 Spain

**Keywords:** Adrenomedullin, Angiogenesis, Tumour growth, PAMP, Xenograft, CLR, RAMP, Tumour microenvironment

## Abstract

Adrenomedullin (AM) is a regulatory peptide whose involvement in tumour progression is becoming more relevant with recent studies. AM is produced and secreted by the tumour cells but also by numerous stromal cells including macrophages, mast cells, endothelial cells, and vascular smooth muscle cells. Most cancer patients present high levels of circulating AM and in some cases these higher levels correlate with a worst prognosis. In some cases it has been shown that the high AM levels return to normal following surgical removal of the tumour, thus indicating the tumour as the source of this excessive production of AM. Expression of this peptide is a good investment for the tumour cell since AM acts as an autocrine/paracrine growth factor, prevents apoptosis-mediated cell death, increases tumour cell motility and metastasis, induces angiogenesis, and blocks immunosurveillance by inhibiting the immune system. In addition, AM expression gets rapidly activated by hypoxia through a HIF-1α mediated mechanism, thus characterizing AM as a major survival factor for tumour cells. Accordingly, a number of studies have shown that inhibition of this peptide or its receptors results in a significant reduction in tumour progression. In conclusion, AM is a great target for drug development and new drugs interfering with this system are being developed.

## Introduction

Adrenomedullin (AM) is a regulatory peptide that was first isolated in 1993 from human pheochromocytoma extracts by Kitamura et al. [1]. These authors found that AM was able to stimulate cAMP production in human platelets and exerted a potent and long-lasting hypotensive activity in rats. AM is synthesized both by tumour cells and by normal adrenal medulla cells, as well as by many other tissues [2]. It is a circulating hormone, although it functions also as a local paracrine and autocrine mediator with multiple biological activities such as vasodilatation, cell growth, regulation of hormone secretion, natriuresis, and antimicrobial effects [2–4].

### Structure of adrenomedullin

Human AM is a small hormone of 52 amino acids. It belongs to the amylin/calcitonin gene-related peptide (CGRP) super-family, which also includes CGRP, amylin and intermedin, also named adrenomedullin 2 [5–7]. The C-terminal tyrosine residue is amidated (-CONH_2_) and AM contains a six amino acid ring formed by an internal disulfide bond between residues 16 and 21. Both structural features are essential for its biological activity.

The three-dimensional structure of AM comprises a central α-helical region, covering approximately one third of its total length, flanked by two disordered segments. The presence of the α-helix at the centre of AM seems to be a general feature of the calcitonin peptide super-family, which is important for the physiology of these peptides and the recognition of their specific receptors [8].

### Adrenomedullin gene expression and release

AM is encoded by the *adm* gene, which has been identified in several mammalian species and is located on human chromosome 11p15.4; consisting of four exons and three introns, with TATA, CAAT and GC boxes in the 5′-flanking region.

The mature AM peptide is derived from preproadrenomedullin, which contains 185 amino acids in humans. After cleaving the 21-residue N-terminal signaling peptide, preproadrenomedullin is converted to proadrenomedullin, which is a precursor of mature AM (amino acids 95-146 of preproadrenomedullin) as well as of another active peptide, proadrenomedullin N-terminal 20-peptide or PAMP (amino acids 22-41 of preproadrenomedullin) [5].

AM production is mostly regulated by oxidative stress and inflammation-related substances such as lipopolisaccharide and inflammatory cytokines such as TNF-α and IL-1, which increase AM secretion rate. There are several binding sites for activator protein-2 (AP-2) and c-AMP-regulated enhancer element. It has also been discovered that there are nuclear factor-Kβ (NF-Kβ) sites on the promoter of the AM [2]. Hypoxia is also a potent inducer of AM expression. This overexpression is mediated by transactivation of the AM promoter by hypoxia inducible factor 1 (HIF-1) transcription factor, as well as by posttranscriptional mRNA stabilization. Hypoxia response elements (HREs) have been found in the promoter of the human *adm* gene [9].

### Metabolism of adrenomedullin

AM is a circulating peptide and it can be found in plasma at a concentration of 2-10 pM in humans. AM is also present in other biological fluids such as urine, saliva, sweat, milk, amniotic fluid and cerebrospinal liquid.

In plasma, AM is specifically bound to adrenomedullin binding protein-1 (AMBP-1), which was later identified as complement factor H [10]. AM bound to complement factor H cannot be detected in plasma, so it is thought that total plasma AM could be higher than reported in most studies. Circulating AM is rapidly degraded with a half-life of 16-20 minutes. Matrix metalloproteinase 2 seems to be responsible for the initial degradation of AM, which is followed by an aminopeptidase [11,12].

### Adrenomedullin receptors

Specific binding sites for AM are located in many cell types and tissues such as the heart, lungs, spleen, liver, vas deferens, kidney glomerulus, skeletal muscle, hypothalamus, and spinal cord, among others. The wide distribution of binding sites for AM is related with its great variety of biological functions. In addition, AM is able to bind to many areas of the brain, providing the anatomical basis for the involvement of AM in the physiology of the central nervous system [13].

The AM receptor contains a member of the 7-transmembrane domain G-protein-coupled receptor superfamily which is named calcitonin receptor-like receptor (CLR). However, CLR needs the presence of modulating proteins with a single transmembrane domain known as receptor activity modifying proteins (RAMP). Three RAMPs have been identified in the human genome: RAMP1, RAMP2, and RAMP3. RAMPs bind to the CLR in the endoplasmic reticulum promoting transport to the plasma membrane [14].

RAMP1 transports CLR to the membrane surface as a mature glycoprotein, and this heterodimer functions as a CGRP receptor [14]. The CLR molecules transported by RAMP2 and RAMP3 are core-glycosilated and function as AM receptors (AMR); CLR/RAMP2 is known as AMR_1,_ whereas CLR/RAMP3 is dubbed AMR_2_ [5,15]. It is hypothesized that residues present in RAMP2 and 3 but not in RAMP1 could be able to alter the pharmacology of CLR and be responsible for making CLR/RAMP2 and CLR/RAMP3 AM receptors [16].

The expression of RAMP isoforms in a particular cell may change between physiological and pathological conditions [17], determining the degree of response to AM and CGRP [18,19]. In physiological conditions the more abundant isoform is RAMP2. The most robust changes in RAMP expression levels coincide with those situations in which plasma AM level is most elevated, as in pregnancy or diseases like sepsis or heart failure. In those situations, there is an elevation in RAMP3 expression, apparently in order to decrease AM responsiveness [18].

### Signal transduction mechanisms

The signal transduction pathways activated by AM vary between species, organs, tissues, and cells. However, there are three main signaling pathways whereby AM exerts its actions: cAMP, Akt, and mitogen activated protein kinase (MAPK)-extracellular signal regulated-protein kinase (ERK).

The main signal transduction pathways activated by AM seems to be the adenylyl cyclase/cAMP system. In many cell types, AM and CGRP receptors are coupled to G_s_ proteins that activate adenylate cyclase and increase intracellular levels of cAMP [5]. In bovine aortic endothelial cells and vascular smooth muscle cells (VSMC) the accumulation of cAMP causes the activation of protein kinase A (PKA) which in turn increases calcium (Ca^2+^) efflux leading to relaxation of the vascular cells [20]. Moreover, it was confirmed that AM can induce Ca^2+^ mobilization independently of cAMP levels. AM activated phospholipase C through its specific receptor and accelerated inositol-1,4,5-P_3_ formation to stimulate Ca^2+^ release from the endoplasmic reticulum intracellular store. In addition, the activation of phospholipase C is also involved in ion channel opening [20,21]. However, other studies have shown that AM administration does not have any effects in intracellular Ca^2+^ concentration and even decreases Ca^2+^ content in cultured human umbilical vein endothelial cells (HUVECs) [22] or in porcine coronary arteries [23]. These results suggest that the regulation of Ca^2+^ mobilization by AM may depend on the cell type and physiological context.

Intracellular Ca^2+^ increases, in response to AM, caused activation of nitric oxide (NO) synthase and NO release leading to relaxation of cardiac myocytes [24]. AM activation of NO pathway has a very important role in the regulation of the cardiovascular system by regulating blood-flow [25], having a cytoprotective action against ischemia/reperfusion injury and against myocardial ischemia-induced arrhythmias in rats [26]. Furthermore, it has been demonstrated that AM inhibited endothelial cell apoptosis through a NO-dependent pathway [27]. Some authors postulate that NO prevents apoptosis by S-nitrosylating caspases [28–30].

AM has been shown to activate the PI3K/Akt pathway in vascular endothelial cells where it regulates many steps such as vasodilation, cell survival, proliferation, migration and vascular cord-like structure formation [31]. The specific role of AM in the multistep process of angiogenesis is regulated via a mechanism that requires the activation of the AMR_1_ and AMR_2_ receptors [32]. AM also acts directly on myocardium by the presence of CLR in myocytes, where it enhances neovascularization, induces cardioprotective effects and exerts antiapoptotic effects through the PI3K-dependent pathway after ischemia/reperfusion [33].

The role of AM in growth and mitogenesis has led to investigate the regulation of MAPK by AM. AM appears to either stimulate or inhibit cell proliferation depending on the particular cell type. AM signalling directly promotes endothelial cell growth and survival through activation of MAPK/ERK downstream signalling pathways [34]. Under serum deprivation, AM promotes DNA synthesis and cell proliferation in VSMCs [35,36]. These responses are mediated by p42/p44 MAPK activation. Interestingly, in glomerullar mesangial cells AM causes an opposite effect by increasing apoptosis during serum deprivation [37]. Activation of MAPK and other kinases such as cAMP-PKA, JNK and protein phosphatase 2A (PP2A) have been proposed to mediate the proapoptotic effect of AM in mesangial cells. On the other hand AM protects malignant cells from hypoxia-induced cell death by up-regulation of Bcl-2 in an autocrine/paracrine manner [38].

### Physiological activities of adrenomedullin

All signal mechanisms in which AM is involved are the basis of this peptide’s extensive repertoire of biological functions such as vasodilation, cellular proliferation, apoptosis modulation or inflammatory regulation, among others.

The main role played by AM in mammalian development has become apparent following the generation of different knockout (KO) models. In *adm* gene KO mice, in which the expression of AM and PAMP are suppressed, the null phenotype is embryonically lethal due to the scarcity of placental vascularization, malformation of the basement membrane in the aorta and cervical arteries, detachment of the endothelial cells from the basement structure, and the presence of edema [39]. Very recent studies have confirmed these results, demonstrating that locally produced AM in the trophoblast binucleate cells of the bovine placenta may play a crucial role in regulation of placental vascular and cellular functions during pregnancy, especially during transition from the mid to late gestation period [40]. KO mouse models in which only AM expression, but not PAMP expression, is affected are also embryonic lethal between embryonic day 14 (E14.5) and embryonic day 15 (E15.5) [41]. Thus, AM may be intimately related with embryonic development and pregnancy [42,43].

A gene-targeted KO model of the CLR gene, *Calcrl*, demonstrates that *Calcrl* is also essential for embryo survival. *Calcrl*^*-/-*^ pups are not viable, the embryos die between E13.5 and E14.5 of gestation and they exhibit a very similar phenotype to AM^-/-^ and AM/PAMP^-/-^ mice [44].

In models of mice lacking RAMP2 the results are similar to the ones shown above. RAMP2^-/-^ embryos die in utero at midgestation due to severe deformation, vascular fragility, severe edema and hemorrhage [45]. Very recent studies with endothelial cell-specific RAMP2 KO mice (E- RAMP2^-/-^) have confirmed that the AM-RAMP2 system is a key determinant of vascular integrity and homeostasis from prenatal stages through adulthood [46].

Surprisingly, a complete absence of RAMP3 has no effect on survival. RAMP3-null mice appear normal until old age (9-10 months), at which point they have less weight than their wild-type littermates [47]. These results provide support to the hypothesis that RAMP2 and RAMP3 have distinct physiological functions in embryogenesis, adulthood, and old age.

To continue with the study of the lack of AM in adult tissues and organisms, tissue-specific conditional KO models have been generated using Cre/loxP technology [48].

In the adult organism, AM has been located in many cell types and in most tissues throughout the body [49], including the nervous system and related structures, cardiovascular system, endocrine organs, digestive tube, excretory system, respiratory system, reproductive tract and integument, among others.

AM has a variety of biological actions which are of potential importance for cardiovascular homeostasis, growth and development of cardiovascular tissues and regulation of body fluid [50–53]. Systemic AM administration has demonstrated that this peptide reduces arterial pressure, decreases peripheral vascular resistance, and increases heart rate and cardiac output [54–57]. Moreover, AM and PAMP function as potent angiogenic agents [58], are necessary to maintain the integrity of the mucous membrane’s microvasculature [46], and promote a faster healing of epithelial wounds [59–61]. AM binds to specific receptors in endothelial cells and elicits endothelium-dependent vasorelaxation mediated by NO [62], endothelium-derived hyperpolarizing factor [63], and/or vasodilatory prostanoids [64].

AM exerts a tight control on renal function and body fluid volume [65,66], regulating the hypothalamic-pituitary-adrenal axis at all levels [67].

AM regulates hormone secretion in many tissues and organs. Levels of this peptide have effects in the hypothalamic-pituitary-adrenal axis as shown above [67]. In addition, AM is synthesized in pancreatic polypeptide-producing F cells of the pancreatic islets and AM receptors are expressed in insulin-producing β-cells [68]. Several studies have shown that endogenous AM tonically inhibits insulin secretion [68,69].

In the digestive system, AM immunoreactivity is widely distributed in the mucosal and glandular epithelia of the stomach, esophagus, intestine, gallbladder, bile duct and acini of the pancreas and salivary glands [70]. AM is a potent inhibitor of basal gastrin-stimulated HCl secretion [71].

AM and its receptors are abundantly expressed in the central nervous system and its cellular components [70,72]. It plays an important role in the regulation of specific blood-brain barrier properties [73], it also increases the preganglionic sympathetic discharges [74], and it exerts several neuroprotective actions against ischemic damage [75]. In addition, relatively recent studies suggest that AM may be involved in the neuroendocrine response to stress and nociception [76,77].

Finally, AM has been found in all epithelial surfaces which separate the external and internal environment and in all body secretions [78]. This wide distribution suggests the possibility that AM has an immunity-related function. It has been proven that both AM and PAMP display potent antimicrobial action against Gram-positive and Gram-negative bacteria [3].

### Adrenomedullin and disease

Elevation of AM levels in plasma has been observed for a variety of cardiovascular disorders. Accumulating evidence supports a compensatory role for AM in heart failure [79] and myocardial infarction [80]. It has been established that plasma AM levels increase in patients with heart failure in proportion to the severity of the disease [81]; and they are also increased during acute phase of myocardial infarction reaching its maximum on day 2–3 and returning to baseline after about 3 weeks [80]. Furthermore, recent studies suggest that plasma AM level is an independent prognostic indicator of heart failure [55,82] and AM exerts a protective action against ischemia-reperfusion injury after stroke [55]. It is well established that AM also protects against ischemia-reperfusion injury in other organs, such as the kidney [83] or the brain [75].

AM was detected in macrophages found within the atherosclerotic plaque [84]. Plasma AM is increased in patients with chronic ischemic stroke and correlates with the extent of carotid artery atherosclerosis [85]. In theory, AM could inhibit atherogenesis due to its inhibitory effect on migration and proliferation of vascular smooth muscle cells, inhibition of endothelial cell apoptosis and anti-inflammatory activity.

Elevation of plasma AM concentration is also observed in patients with primary arterial hypertension and is higher in individuals with complications of hypertension, such as left ventricular hypertrophy and nephrosclerosis [86]. It is suggested that the up-regulation of cardiac AM system in hypertension is a protective mechanism decreasing myocardial overload due to vasodilatory and natriuretic properties of AM, as well as limiting further myocardial hypertrophy and remodelling [87].

Plasma AM concentration is increased, whereas urinary AM excretion is decreased in various types of glomerulonephritis [88]. In addition, plasma AM progressively increases in patients with chronic renal failure [89].

In septic shock patients, a marked elevation of AM blood levels has been reported, probably as a defensive action [90–92]. However, excessive AM release during septic shock may provoke adverse effects such as hypotension which may threaten the patient’s life [3].

AM also plays a role in primary and secondary pulmonary hypertension. Very recent studies in rats with pulmonary hypertension induced by high blood flow suggest that AM exerts a protective action in the development of this pathology, by inhibiting pulmonary procollagen synthesis and alleviating pulmonary artery collagen accumulation [93].

Furthermore, AM has emerged as a novel and promising therapy for digestive pathologies related with inflammation such as gastric ulcers [94] and inflammatory bowel diseases [95,96]. This is closely related with the local and systemic anti-inflammatory actions that AM is able to exert [97,98]. For example, it has been demonstrated that AM inhibits the secretion of pro-inflammatory cytokines when it is released to the medium by peripheral blood monocytes [5] and plays a role in the evolution of Th1/Th2 cytokines balance, decreasing pro-inflammatory cytokines levels (IL-6, IL-10, TNF-α, IFN-γ) [99–101]. In addition to the regulatory role on immune cells, AM also decreases endothelial permeability, thus reducing the formation of inflammatory exudates [5].

The existence of AM in pancreatic islets and its inhibitory effect on insulin secretion suggest that AM may be involved in the pathogenesis of diabetes mellitus. In type 1 diabetes, plasma AM is increased only in patients with microangiopathy. Increased AM may result from endothelial activation and/or impaired renal clearance in subjects with diabetic nephropathy [102]. This suggests that increased AM in type 1 diabetes is a consequence of the disease rather than a causal agent. In humans, the levels of circulating AM are clearly elevated in patients with type 2 diabetes when compared to normal controls [103,104]. In addition, AM has emerged as a possible biomarker for early diagnosis in pancreatic cancer-induced diabetes [105].

AM is also involved in many eye pathologies. AM levels in the plasma, vitreous fluid samples and fibrous membrane tissues are all significantly elevated in patients with proliferative diabetic retinopathy compared with control subjects [106]. AM concentration in vitreous fluid is markedly increased in patients with proliferative vitreoretinopathy, the most common complication of retinal detachment originating from the proliferation of retinal pigment cells [107].

### Adrenomedullin levels in cancer patients

There are now many studies that show an association between AM expression and cancer. Initially, these were predominantly studies where plasma AM concentrations were measured in patients suffering from different tumour types and compared with healthy patients. These tumours included bronchial neuroendocrine tumour, clear cell renal cell carcinoma, midgut tumour, osteosarcoma, pancreatic adenocarcinoma, pancreatic insulinoma, aldosterone-producing adenoma, pheochromocytoma, pituitary adenoma, and plexiform neurofibroma [108–118] and there were significant increases of AM levels in all cancer patients.

Interestingly, in patients with osteosarcoma, insulinoma, pheochromocytoma, and primary aldosteronism due to adenoma, elevated blood AM levels decreased following surgery and returned to normal [109,111,113,114], indicating that the tumour was the main source of these excessive AM levels. However, plasma AM levels of patients after 4–5 weeks of surgery of clear cell renal cell carcinoma and of other kidney tumours were similar to pre-surgery levels. Therefore, plasma AM is not suited as a tumour marker for this disease [118].

Nowadays with the accessibility of molecular techniques, AM mRNA and/or protein expression have also been determined in different tumour types and compared with normal tissue, normal-looking tissue adjacent to the tumour, or with other related no tumoural and pathological tissues. Several clinical studies suggest that AM is over-expressed in numerous tumours including colorectal cancer, bladder urothelial cell carcinoma, chromophobe renal carcinoma, clear-cell renal carcinoma, osteosarcoma, pancreatic adenocarcinoma, insulinoma, ovarian carcinoma, endometrial cancer, leiomyoma, glioma, glioblastoma, neuroblastoma, ganglioneuroblastoma, pituitary adenoma (ACTH-secreting), somatotropinoma, astrocytoma, hepatocellular carcinoma, non-small cell lung carcinoma, squamous cell carcinoma, adenocarcinoma of the lung, bronchial neuroendocrine carcinoma, midgut neuroendocrine carcinoma, pheochromocytoma, aldosterone-producing adenoma, breast cancer, intraocular or orbital tumours, and melanoma, as shown in Table 1 [108–110,116,118–144]. However, AM expression in the anterior pituitary is diminished in tumours as compared to the normal gland [145]. On the other hand, as described by Letizia et al. [112], blood AM concentrations in control patients was low as compared to the setting of Cushing disease due to pituitary adenoma [112]. These data could be interpreted by an increased release of AM from the secretory granules of the pituitary into circulation. In prostate, no difference on the expression of AM was detected between benign epithelial cells adjacent to prostate adenocarcinoma lesions and tumour [146].

**Table 1:** **Expression of AM and AM receptors in tumours and their role on disease progression**

**Cancer type**	AM in plasma*	**AM expression**	**Receptor expression**	DP**	**References**
Breast carcinoma	presence	Prot		lymph node metastasis	[138,144]
Bladder urothelial cell carcinoma		>mRNA/Prot			[120]
Chromophobe renal carcinoma		>mRNA			[121]
		<Prot			
Clear-cell renal carcinoma		>mRNA	CLR and RAMP2		[118,121]
		Prot			
Colorectal carcinoma		>mRNA	>CLR, RAMP2, RAMP3	progression	[119,147,148]
		>Prot			
Midgut tumour	>			progression	[108]
Anaplastic astrocytoma		<mRNA			[128,131]
Glioma		>mRNA		progression	[127]
Glioblastoma		>mRNA	CLR, RAMP2 and RAMP3		[121,128,131]
Hepatocellular carcinoma		>mRNA		invasion and progression	[132,133]
		>Prot			
Intraocular or orbital tumours		>mRNA			[139]
Leiomyoma		>Prot			[126]
ganglioneuroblastoma		>Prot			[129]
Neuroblastoma		>mRNA		differentiation	[129,143]
		>Prot			
Bronchial neuroendocrine tumour	>			progression	[108]
Small cell lung carcinoma		<mRNA			[134,141]
Non-small cell lung carcinoma		mRNA immunoreactivity was essentially weak			[134,141]
Squamous cell carcinoma of the lungs		<mRNA			[134,141]
Adenocarcinoma of the lung		mRNA			[134,141]
Osteosarcoma	>	>mRNA / Prot		metastasis	[109]
Ovarian carcinoma		> mRNA / Prot		over-all survival	[123,124,142]
				Positive Prognostic Factor	[149]
Endometrial carcinoma		>mRNA		progression	[125,150]
		<Prot			
Pancreatic adenocarcinoma	>	>AM &CLR mRNA / Prot	CLR, RAMP1 and RAMP2		[110,116,122,151]
Pancreatic insulinoma	>	>Prot			[110,111]
Adrenocortical tumours	>	mRNA			[113,135,136]
		Prot no detected			
Pheochromocytoma	>	mRNA	CLR, RAMP1, RAMP2 and RAMP3		[114,117,129,135–137,152]
		>Prot			
Pituytary adenomas	>	>Prot		progression	[112,130,136,145]
Plexiform neurofibroma	>			biomarker of transformation	[115]
Somatotropinoma		>mRNA			Knerr *et al.*, [131])
Prolactinoma		mRNA			
meningiomas		mRNA			
Prostate		mRNA		high Gleason scores	[146,153]
adenocarcinoma		Prot			
Skin carcinomas		Prot	> CLR, RAMP2, and RAMP3		[78,140]

*>:higher plasma AM concentration in cancer patients than in healthy controls.

**DP: Correlation of AM with disease progression.

In most tumours a high AM mRNA expression correlated with high protein expression. However, in endometrial cancer tissues and chromophobe renal carcinoma, although AM mRNA levels were high, the protein expression was mild [121,125], indicating complex post-transcriptional regulation.

Furthermore, in some tumours it is possible to correlate plasma levels and expression of AM with disease progression. Plasma levels and AM expression of bronchial neuroendocrine carcinomas and midgut neuroendocrine carcinomas correlate with tumour progression [108]. In breast cancer, AM plasma concentrations correlate with the presence of lymph node metastasis [144]. AM expression is highly correlated with the degree of malignancy and metastasis of osteosarcoma [109]. In patients with leiomyomas, high AM expression is associated with increased vascular density [126]. Epithelial ovarian cancer patients with high AM expression showed a higher incidence of metastasis, larger residual size of tumours after cytoreduction, and shorter disease-free and overall survival time [123]. AM gene expression levels may play a key role in the biology of epithelial ovarian cancer and may define a more aggressive tumour phenotype [124]. However, recent studies performed in ovarian cancer patients by Baranello et al. found high expression levels of AM as a positive prognostic factor [149]. In hepatocellular carcinoma, AM expression was positively correlated with invasion and progression [132,133]. Elevated AM mRNA was associated with high Gleason scores in prostate cancer [153]. High AM mRNA levels were associated with an increased risk of relapse in patients who underwent surgery for localized clear cell renal and colorectal carcinoma [121,147]. In colorectal carcinoma, AM mRNA levels are also a significant factor for poor prognosis and incidence of liver metastasis [147]. The expression of AM is associated with melanomagenesis in melanoma patients [140]. AM mRNA in neuroblastoma is linked to tumour differentiation [143]. The correlation of AM expression and the grade of glioma supports the hypothesis that AM may participate in the progression of the tumour [128].

There is significant evidence for the association of the expression of AM and its receptors with cancer. AM and CLR mRNA levels were higher in pancreatic adenocarcinoma tissues compared to normal pancreatic tissues [116]. The expression levels of AM, CLR, RAMP2 and RAMP3 in human melanoma were higher than in control tissues [140]. Tissue microarray analysis of human colorectal tumours revealed a clear increase of AM, CLR, RAMP2, and RAMP3 staining in lymph nodes and distant metastasis when compared with primary tumours [119]. Recently, new cancer risk markers are being developed. Cheung et al demonstrated that carriers of a single nucleotide polymorphism (SNP), rs4910118, had significantly lower levels of circulating AM than homozygotes for the more common allele [154]. In agreement with this, Martinez-Herrero et al. described that carriers of the rs4910118 SNP have a 4.6-fold lower risk of developing cancer than homozygotes for the major allele [155].

Midregional -proadrenomedullin (MR-proAM) is a stable and reliable surrogate marker for AM release levels. MR-proAM was measured in plasma from persons without cancer prior to the baseline exam. During the follow-up median period of 14 years diverse cancer events occurred. In this context, MR-proAM predicts later development of cancer in males, particularly in younger males [156].

### Adrenomedullin and tumour microenvironment

There is an increasing body of evidence suggesting that malignant growth encompasses several processes including increase in growth signals, angiogenesis and metastasis, inhibition of apoptosis, and others [157–159].

Although AM does not cause cancer by itself it can promote its advance through different mechanisms. In addition, known carcinogens such as cigarette smoking can increase AM expression through activation of aryl hydrocarbon receptor (AHR), and blockade of AM can decrease tobacco-induced tumour growth [160]. AM has been shown to be strongly up-regulated in several different tumour types, especially when subjected to hypoxic environments. AM is involved in tumour initiation and progression by promoting cell proliferation, angiogenesis, change of phenotype, and the inhibition of apoptosis [161–164]. In the last years, numerous studies have appeared showing a relation between AM expression and cancer. In most of them, the expression (either mRNA and/or protein) of AM has been compared between normal tissue and different tumour types. In general, the reports show that AM is over-expressed in tumours such as renal cell carcinoma, some endocrine-related tumours, hepatocellular carcinoma, non-small cell lung carcinoma, and others [19,132,134,165–168]. Interestingly, there are some reports of decreased expression of AM in human pituitary adenomas in comparison with nontumoural adenohypophyses [145].

### Adrenomedullin expression in cancer cells and its role on malignant growth

Over the last years, numerous authors have reported the regulatory properties that AM possesses on the proliferation of a wide variety of cancer cells. In 1996, Miller and cols analyzed the expression of AM mRNA by RT-PCR in 20 human normal tissues and 48 tumour cell lines [166]. The authors found that 95% of normal and tumour cells expressed the mRNA for the peptide [166]. Tumour cell lines evaluated included small cell lung carcinomas, non-small cell lung carcinomas, breast, nervous system (glioblastoma, neuroblastomas), ovarian, prostate, adrenal, chondrosarcoma, and chronic monocytic leukemia [166]. These data have been replicated on different tumour cell lines such as pancreatic cell lines (PANC1, L3.6, HPAFII, SU86.86) [110,116,169], gliomas [170,171], prostate cancer cell lines [162,172–175], ovarian cancer [149,176,177], osteosarcoma [109], renal carcinoma [121,178], multiple myeloma [179], bladder urothelial cell carcinoma [120], pituitary adenomas [145], colorectal cancer [119,147,148], breast cancer [144], endometrial cancer [38], hepatocellular carcinoma [132] and others.

It is noteworthy to mention that, in some tumours, RAMP3 is expressed alongside RAMP2 while in others only RAMP2 is present. In renal tumours, for instance, RAMP2 was expressed in the tumour cells themselves, while RAMP3 elevation was found in inflammatory cells associated with the tumour, highlighting the importance of the interaction of the tumour with the microenvironment [121].

Interestingly, Lombardero and cols reported that the expression of AM measured by immunohistochemistry in various hormone-secreting pituitary adenomas was found to be diminished as compared to nontumoural adenohypophyses [145], although this fact may represent a faster disregulated secretion of the peptide to the blood stream.

### Adrenomedullin expression is increased under hypoxic conditions

Focal areas of hypoxia are inherent to the environment of solid tumours [180,181]. Decreased oxygen availability is one of the driving forces of cancer survival and progression. When tumour cells are exposed to hypoxic conditions, an oxygen-sensing mechanism, based on the hypoxia-inducible factor-1 (HIF-1), mediates the expression of a group of genes that help tumour cells to survive [9,182,183]. Several studies have addressed the regulation of AM (and its receptors) expression under hypoxic conditions in a variety of tumour tissues and cell lines. The first authors to report an induction of AM in a tumour cell line exposed to hypoxia were Nakayama and cols in 1998 [184]. Human colorectal carcinoma cells exposed to a reduced oxygen tension showed a time-dependent increase in AM mRNA and peptide expression. Later on, the hypoxia-induced upregulation of AM expression was described in a variety of human tumour cell lines from lung, breast, ovary, prostate, bone, blood [9], multiple myeloma [179], bladder urothelial cell carcinoma [120], colorectal carcinoma [119,147,148] and hepatocellular carcinoma [132], among others. The first proof that this increased expression was mediated by HIF-1 was provided by Garayoa and cols [9] using HIF-1α and HIF-1β knockout cell lines.

As an example to illustrate the effect of AM in hypoxic environments we can compare the role that AM plays in the pathophysiology of pilocytic astrocytomas and glioblastomas. Pilocytic astrocytoma is a slowly growing tumour where preexisting blood vessels are sufficient to provide enough oxygen and to ensure tumour growth [171]. Glioblastoma, however, is a rapidly growing tumour where normal blood supply is not sufficient, leading to necrosis and hypoxia [171]. Real-time quantitative RT-PCR was used to study expression of AM, RAMP2, RAMP3, and CLR in pilocytic astrocytoma and glioblastoma. Interestingly, although there were not differences in RAMP2 or RAMP3 expression, AM mRNA expression was induced in glioblastoma whereas it was barely detectable in pilocytic astrocytoma when subjected to hypoxic conditions. Furthermore, AM and VEGF mRNA expression were highly correlated, supporting the view that AM may function as an autocrine/paracrine growth factor for glioblastoma cells subjected to hypoxia [171].

### Adrenomedullin is a survival factor for tumour cells

Adrenomedullin is able to reduce apoptosis of both endothelial and tumour cells. AM-overexpressing endometrial tumour cells, prostate cancer cells, or breast carcinoma cells present reduced levels of proapoptotic proteins such as fragmented PARP, Bax, and activated caspases, resulting in lower level of induced-apoptosis compared with control cells [165,172,185]. However, the up-regulation of AM in tumours can be used to design strategies to treat these types of cancer. For instance, AM expression up-regulated the expression of IL13 receptor α2 which can be used to increase the sensitivity to IL13PE cytotoxin (consisting of IL-13 and a truncated form of Pseudomonas exotoxin) [175].

AM can stimulate cell growth and inhibit apoptosis in a variety of tumour cells, including prostate cancer [172,174,175], ovarian cancer [149,176], osteosarcoma [109], renal carcinoma [178], bladder carcinoma [120], breast cancer [186], colorectal cancer [119,148], gliomas [170], and hepatocellular carcinoma [132].

Thus, when AM was overexpressed in the endometrial cancer cell line RL95.2 a marked growth increase was seen in response to hypoxia-induced apoptosis [38]. Similarly, T47D and MCF-7 breast tumour cell lines challenged with serum-free conditions were able to maintain cell proliferation only in the presence of AM [165]. Also, the implication of AM in the survival of U87 glioblastoma cells was demonstrated by intratumoural administration of an anti-AM antibody in xenografted mice which resulted in a 70% decrease in xenograft weight and density of tumour vessels.

The role of AM in prostate cancer cell pathophysiology seems to be controversial. Depending on the cell line used (PC-3, DU145, or LNCap) and the insult (etoposide or serum deprivation) the effect of AM on proliferation/apoptosis differs [172,173]. After serum deprivation, AM prevented apoptosis in DU145 and PC-3 cells, but not in LNCaP cells [172,173]. However, after treatment with etoposide, AM prevented apoptosis in PC-3 and LNCaP cells, but not in DU145 cells [172,173]. Surprisingly, although PC-3 prostate cancer cells over-expressing AM generated smaller tumours in vivo when injected in nude mice [172], blockade of AM by an specific antibody in DU145 prostate cancer cells induced a clear regression of tumour growth and metastasis in a xenograft mouse model [174].

As noted above, the presence of AM peptide, as well as AM receptors, has been described in ovarian cancer cells [178]. Silencing of the AM gene inhibited the proliferation and increased the chemosensitivity of HO8910 cells by downregulation of Bcl-2 and p-ERK, as described for other cancer cell types [176]. However, other authors have reported that AM effect in ovarian cancer cells is weak as revealed by proliferation assays and cell cycle analysis performed under stressing conditions, such as serum starvation and/or hypoxia [149]. Baranello and cols found that AM was a survival factor for HEY cells but not for A2780 or OVCAR-3 ovarian cancer cells. Furthermore, a clinical study revealed that high expression of AM was linked to a positive outcome, suggesting that the use of AM antagonists could be deleterious in the treatment of ovarian cancer patients [149].

Although the expression of AM, CLR, RAMP1, and RAMP2 mRNA has been reported in several pancreatic cancer cells, RAMP3 mRNA expression could only be found in 1 of 5 cell lines studied [116]. These observations, and the strong colocalization of CLR with RAMP1/RAMP2 but not with RAMP3, indicate that RAMP1/2 but not RAMP3 are the main coreceptors for CLR in pancreatic adenocarcinoma [116]. Intratumoural injection of AM antagonist peptides or transfer of naked DNA encoding AM antagonists induce the regression of a pancreatic cancer cell line and a breast cancer cell line in a mouse xenograft [169]. In addition to its role regulating proliferation/apoptosis, the blockade of AM action seems to also involve a reduction in tumoural neovascularization, which is entirely inhibited in AMA (adrenomedullin antagonist)-treated mice [169].

The role of AM in cell growth and invasion in human colorectal tumours has also been explored recently. Human colon carcinoma cells (HT-29, HCT116, DLD1, and SW480) express AM, CLR, RAMP2, and RAMP3, and the expression of AM is increased under hypoxic conditions [119,147,148]. Addition of synthetic AM to tumour cells in culture stimulated cell proliferation and invasion which could be reversed by co-incubation with an AM antibody or an AM antagonist [119,148]. Furthermore, AM antibody treatment significantly reduced the growth of HT-29 tumour xenografts in mice [119]. These data seem to correlate well with clinical data where AM has been described as an independent prognostic factor for colorectal cancer [147].

In recent years numerous xenografted tumour models have been used to provide new insights in the understanding of AM’s role in tumour growth in vivo. Interestingly, vascular density or directed growth of blood vessels measured in these xenograft models correlates well with AM expression. Thus, human endometrial, breast, lung or pancreatic tumour cell lines overexpressing AM show an increase in blood vessel density [38,165,169,187,188], while colorectal, prostate, and renal carcinoma cells with decreased AM availability resulted in blood vessel density reduction [119,148,174,189]. Similar results were obtained when xenografting human glioblastoma cells, who express high basal levels of AM. Both density of blood vessels and cell growth were decreased when an antibody against AM was administered intratumourally [128].

All these data taken together support the idea that AM functions as a potent autocrine/paracrine growth factor for tumour cells and demonstrate that reduction of endogenous AM, either pharmacologically or by gene therapy, can potentially impair tumour growth in vivo. The collective findings point out that the autocrine loop formed by AM and its receptors plays a major role in tumour formation and progression, and that it may be a target for new treatments against malignant diseases.

### Adrenomedullin in extra-tumoural components

Stromal factors interact with cancer cells to establish a microenvironment that supports tumour growth and survival. AM is an autocrine/paracrine peptide produced by stromal and cancer cells to support such a microenvironment [149]. AM enhances blood and lymphatic angiogenesis, providing necessary nutrients and oxygen to the tumour cells to grow and, eventually, to disseminate [9,34,149]. The main sources of AM are the vascular endothelium and, usually, the tumour cells themselves, although other types of cells such as mast cells, macrophages and fibroblasts can also produce the peptide [138,140]. The role of AM in tumourigenic angiogenesis has been studied using several in vitro, xenograft, and knockout mouse models [38,165,168,185]. AM can regulate the tumour microenvironment by promoting proliferation and migration of endothelial cells, reducing the activity of the immune system by reducing cytokine secretion [3], and inhibiting the complement pathway [168,183]. Experiments using AM knockout mice demonstrated that AM is essential for vascular morphogenesis in normal animals [39,188,190], as well as in tumours. In fact, AM is not only able to enhance bone marrow-derived mononuclear cell differentiation into endothelial cells but also it is important for the formation of mature vessels [191].

### Adrenomedullin and cancer treatment

Several strategies have been proposed to inhibit AM-induced tumour growth, including AM mRNA rybozyme which modulates AM expression, and other approaches targeting AM binding to its receptor for example anti-AM blocking antibodies, small nonpeptide molecules, receptor antagonists, truncated peptides, e.g. AM22–52 (AMA) and PAMP12-20 [167,192].

Various studies using human tumour xenografts in immuno-deficient mice have shown that lowering AM levels reduces tumour growth. For example, growth of sarcoma tumours was slower when injected in heterozygotic AM knockout mice as compared to their wild type counterparts. In addition, treatment of tumours with a competitive inhibitor of AM (AMA) resulted in tumour reduction [193]. Besides, tumour weight was reduced following intra-tumoural injection of an AM antagonist (AMA) in mouse models of pancreatic [169,187], mammary [169], and skin [140] cancer cell growth. Furthermore, a single intra-tumoural or intra-muscular transfer of naked DNA-encoding AMA suppresses renal cell carcinoma growth [189].

Moreover, targeting AM receptors (AMR) with systemic delivery of neutralizing antibodies inhibits growth of human tumour xenografts in mice. Antibodies against AMR significantly reduced the growth of glioblastoma [53,128], lung [53], prostate [174], colon tumours [53,119], and melanoma [140] growth in vivo. Although some authors have raised concerns about the specificity of the antibodies against the receptors [194], the original authors performed pre-absorption tests that resulted in successful band suppression showing at least immunological specificity against synthetic peptides used as antigens [32,53]. Nevertheless, more studies from other laboratories are needed to completely characterize these antibodies and to confirm originally obtained results.

Other strategies targeting AM includes RNA interference that reduced the growth of human bladder urothelial cell carcinoma [120]. In addition, the peptide fragment PAMP (12-20) diminished tumour growth in a xenograft model using lung carcinoma [195].

Obviously none of these potential treatments has undergone pre-clinical and clinical testing and nothing is known about potential side effects and/or toxicity in humans. Therefore we must be careful with the interpretation of previous data until clinical trials have been performed.

## Conclusion

All these studies support the idea of AM as a survival factor for tumour cells, that can be produced either by the tumour itself or by a number of stromal cells surrounding the tumour. In general, AM expression is upregulated by hypoxia, a common occurrence in tumours, and the excessive production of this peptide results in poorer prognosis for the patients. Therefore, new therapies based on the blockade of the AM autocrine/paracrine system are been developed and some of them are very effective in animal models. It remains to be seen whether this efficacy would persist in clinical trials.
